# Acute Bacterial Hemorrhagic Pyelonephritis in a COVID-19 Patient With a History of Hypothyroidism: A Case Report

**DOI:** 10.7759/cureus.21730

**Published:** 2022-01-30

**Authors:** Omar Shazley, Ameer Shazley, Moudar Alshazley

**Affiliations:** 1 Basic Sciences, Saint James School of Medicine, Park Ridge, USA; 2 Emergency Medicine, Internal Medicine, Santa Rosa Medical Center, Pensacola, USA

**Keywords:** kidney stone, ureteral obstruction, cytokine storm syndrome, hypothyroid, sars-cov-2, covid-19, pyelonephritis

## Abstract

Since its initial reporting in December 2019, the novel coronavirus SARS-CoV-2 has emerged as a global health problem after its official declaration as a pandemic by the World Health Organization, with an estimated 346 million cases and over 5.9 million fatalities as of January 22, 2022. Studies on the prevalence of COVID-19 among severe cases have shown that comorbidities and risk factors such as obesity, increased aging, and chronic cardiovascular and respiratory diseases play a role in the severity of SARS-CoV-2 infections. The interactions between such factors and their involvement with the progression of infection and mortality remain unclear.

While it is known that SARS-CoV-2 damages the lungs, various morbidities such as acute kidney disease and thyroid dysregulation have recently emerged in symptomatic COVID-19 patients. Conditions that alter thyroid hormones, which play a critical role in regulating metabolic pathways, have a role in the level of infectivity of the SARS-CoV-2. The capability of the SARS-CoV-2 to invade and affect any organ system is dependent on its access to the angiotensin-converting enzyme II (ACE2) commonly expressed among various host cells. This binding puts any system at high risk of direct viral injury, inevitably creating an excessively high concentration of anti-inflammatory mediators and cytokines to predispose COVID-19 patients to a state of severe immunosuppression.

This case report describes a 62-year-old female who tested positive for COVID-19, with a medical history of hypothyroidism, who presented with a unique combination of acute bacterial hemorrhagic pyelonephritis and ureteral obstruction. She experienced intermittent dysuria, urinary urgency, and hematuria over the past five days. She developed chills, diaphoresis, nausea, and vomiting after administering acetaminophen for her headache. Ageusia and anosmia accompanied her respiratory illnesses despite receiving the Pfizer double dose vaccine six months before her arrival. A computerized tomography (CT) scan revealed severe to moderate inflammation surrounding the enlarged kidney with a 1 mm ureteral stone. Blood and urine cultures showed the growth of *Escherichia coli* gram-negative bacilli. Chest X-rays displayed a patchy appearance in the right infrahilar airspace, reflecting atelectasis in part for the diagnosis of COVID-19 with additional laboratory findings of profoundly elevated C-reactive protein, fibrinogen, and d-dimer levels. Abdominal CT scans revealed a hemorrhagic ureteral obstruction and massive swelling of the renal parenchyma persistent to pyelonephritis and hydronephrosis.

## Introduction

Coronavirus disease 2019 (COVID-19) was declared a pandemic by the World Health Organization on March 11, 2020. Although common symptoms of COVID-19 are related to the respiratory system, severely infected patients are more prone to the progression of respiratory failure and multiple organ injuries, including the kidneys, liver, heart, and gastrointestinal tract [1]. In previous cases, risk factors that lead to more severe SARS-CoV-2 infections have been identified as comorbidities such as obesity, increased aging, cardiovascular disease, type 2 diabetes, hypertension, and pre-existing conditions, particularly cardiovascular and metabolic conditions [2]. The interactions between such factors and their roles in the progression of infection and mortality remain unclear.

The secretion of thyroid hormones maintains normal human physiology and homeostasis. Thyroxine (T4), along with triiodothyronine (T3), is influenced by the concentration of thyroid-stimulating hormone (TSH) secreted by the pituitary gland. With the use of thyroid function tests for COVID-19 hospitalization, a study conducted on patients with COVID-19 displayed remarkably decreased levels of T3 and TSH in comparison to the levels found in healthy patients [3]. Reduced serum levels of T3 and TSH demonstrate the severity of COVID-19 by the hypothalamic-pituitary-thyroid axis becoming dysfunctional [4]. This influences the prevalence of subclinical hypothyroidism to increase and causes significant changes in renal functions with reduced low glomerular filtration rate (GFR) and decreased sodium reabsorption in the urine to cause hyponatremia [5].

Acute kidney infection (AKI) has been observed in 21-59% of hospitalized patients with severe cases of COVID-19 in association with high mortality [6]. An analysis of 2,600 admitted patients for COVID-19 showed a high association with AKI after adjustments for demographics, comorbidities, and laboratory results [6]. The incidence of thrombotic and vascular complications in ICU patients with COVID-19 has increased, resulting from dysfunctional endothelium with abnormal coagulation [7]. Complications related to these abnormalities occur up to 31% of ICU patients with severe COVID-19 infection [7]. 

In this case, we present a unique combination of bacterial hemorrhagic pyelonephritis and ureteral obstruction due to ureteral stone in a patient with a history of hypothyroidism diagnosed with SARS-CoV-2 infection. The ureteral stone and bacteremia caused the hydronephrosis and pyelonephritis; however, the susceptibility of a unique COVID-19-related etiology concerned the notable decrease in the patient’s thyroid hormone levels, consistent with the exact time frame of when her flu-like symptoms began. This occurrence warrants a further review of the understudied relationship between SARS-CoV-2 infection and thyroid hormone alterations that may pose a risk factor for the pathogenesis of renal injury.

## Case presentation

A 62-year-old female presented to the emergency room with a fever, nausea, vomiting, and diarrhea for the past five days. She had been experiencing constant, severe pain localized to the left costovertebral angle (CVA) associated with recurrent dysuria, urinary urgency, and gross hematuria. Associated symptoms include shortness of breath and a cough. The pain emerged while she was asleep, and she rated it a 10/10. She stated there were no alleviating factors but noted movement as an aggravating factor. The patient explained that her continuous vomiting was worsened by oral intake and improved with ondansetron. Hours before experiencing shakiness, chills, and sweats, the patient experienced flu-like symptoms with a headache, from which she felt temporary relief after taking acetaminophen. A transient fever accompanied her symptoms, with ageusia and anosmia. This made the patient suspect possible COVID-19 exposure following a social gathering where fellow patrons displayed similar flu-like symptoms. She noted she and her husband had received the Pfizer double dose vaccine five months prior. Her medical history was remarkable for hypothyroidism and hiatal hernia. She was a non-smoker and denied a history of intravenous drug abuse or recent travel. Past surgical history revealed fundoplication and hysterectomy. The only medication she had taken was her prescribed levothyroxine, 25 μg daily for her longstanding hypothyroidism.

At the time of presentation, the patient had a body temperature of 102.7 °F, a blood pressure of 133/83 mmHg, a heart rate of 140 beats per minute, and a respiration rate of 21 breaths per minute. Supplemental oxygen therapy was provided with the patient’s O_2_ saturation at 89%. The physical examination revealed an alert, oriented woman who appeared well-nourished and well-developed; her cardiac exam was remarkable for sinus tachycardia with regular S1 and S2 but no rubs, gallops, or murmurs; her lungs were remarkable for scattered rhonchi with no cyanosis, clubbing of fingers, or kyphosis; abdominal tenderness in the left lower quadrant; positive left costovertebral angle tenderness; and +2 pulses in all distal extremities. 

**Figure 1:**
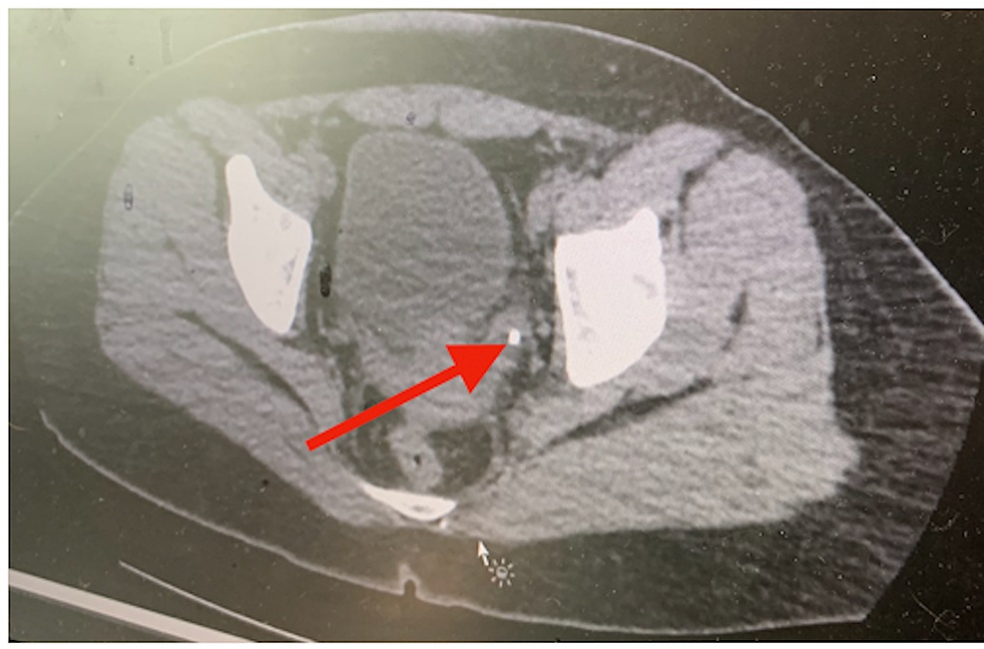
Non-enhanced axial CT scan images of the abdomen and pelvis demonstrate a calcific density at the left ureterovesical junction (red arrow).

**Figure 2:**
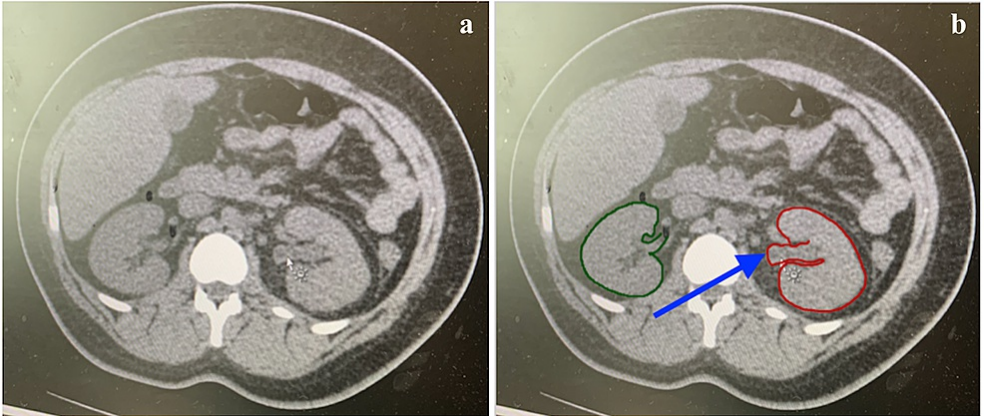
Non-enhanced axial CT scan images of the abdomen and pelvis at presentation. (a, b) Moderately inflamed enlarged left kidney with perinephric edema. (b) Displays the enlarged left kidney by inflammation (red circle) and a dilated intrarenal collecting system (blue arrow).

**Figure 3:**
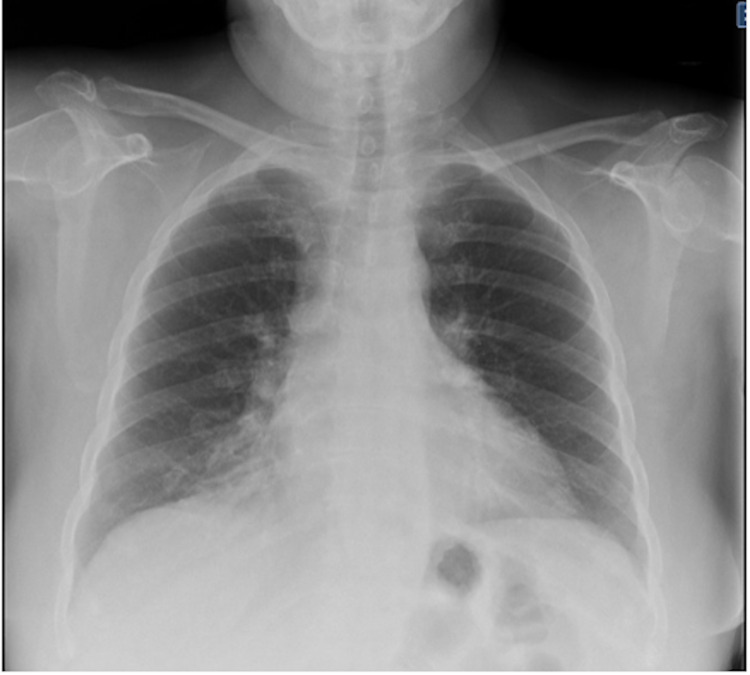
Patient’s initial emergency department chest radiograph. Bilateral airspace consolidations with no acute osseous abnormalities are shown.

Laboratory parameters revealed elevated serum creatinine levels of 125 μmol/L and C-reactive protein of 16.4 mg/L with a decreased eGFR (MDRD) of 62 mL/min/1.73 m² (Table 1). A lowered serum sodium level of 102 mEq/L and potassium of 2.9 mEq/L were measured. The patient’s urine was positive for leukocytes, proteins, erythrocytes, and bacteria. A complete blood count showed leukocytes of 13.9 × 109/L, absolute lymphocytes of 0.4 (103/µL) of blood, and a platelet count of 121,000 per mm³. The patient appeared to have slight anemia with decreased hemoglobin (11.3 g/dL), red blood cell count (3.22 million cells/µL), and iron (53.4 µg/dL). Notable abnormal lab results showed an elevated level of d-dimer at 0.8 μg/mL and fibrinogen at 461 mg/dL (Table 2). Contrast-enhanced computed tomography (CT) revealed a 1 mm ureteral stone in the left proximal ureter near the left pelvis (Figure 1). The findings of moderate inflammation surrounding an enlarged left kidney and parenchymal thickening were consistent with pyelonephritis and hydronephrosis (Figure 2). Ground-glass opacities were found in the right infrahilar region and peripheral basal areas of the lung (Figure 3).

**Table 1:** Laboratory parameters of the patient at the time of arrival and discharge. g/dL = grams per deciliter, L/L = liter of cells per liter of blood, per mm = per cubic millimeter, cells × 10^9^/L = cells per microliter, μmol/L = micromole per liter, mL/min/1.73m^2^ = milliliter per minute per 1.73 m^2^, μg/mL = microgram per milliliter, mg/dL = milligrams per deciliter, mEq/L = milliequivalents per liter, mmol/L = millimoles per liter, mg/L = milligrams per liter, 10^3^/µL = thousands of cells per microliter, million/mm^3 ^= million cells per cubic millimeter, μg/dL = micrograms per deciliter, % = percentage.

Laboratory analysis	Reference value	Day 1	Day 11
Hemoglobin (g/dL)	12.0–16.0	11.3↓	12.9
Hematocrit (L/L)	0.36–0.47	0.38	NA
Platelet count (per mm^3^)	150,000–350,000	121,000↓	205,000
Leukocytes (cells × 10^9^/L)	4.0–10.0	13.9↑	9.8
Creatine (μmol/L)	50–95	125↑	67
eGFR (CKD-EPI) (mL/min/1.73m^2^)	>83	62↓	102
D-dimer (μg/mL)	<0.5	0.8↑	0.38
Calcium (mg/dL)	8.6–10.3	8.5	8.8
Sodium (mEq/L)	135–145	102↓	139
Potassium (mEq/L)	3.5–5.0	2.9↓	4.1
Glucose (mg/dL)	70–100	72	NA
Chloride (mmol/L)	96–106	96	103
CRP (mg/L)	<10	16.4 ↑	6.78
Fibrinogen (mg/dL)	200–400	461 ↑	232
Absolute lymph (10^3^/µL)	1.50–4.00	1.1 ↓	NA
Red blood count (million/mm^3^)	4.20–5.40	3.22 ↓	4.45
Iron (μg/dL)	60–140	53.4 ↓	67.8
Segmented neutrophil (%)	45–75	87.9%↑	72%

**Table 2:** Thyroid hormone parameters of the patient at the time of arrival and discharge. mIU/L = milli-international units per liter, ng/mL = nanograms per milliliter, pg/mL = picograms per milliliter, ng/dL = nanogram per deciliter.

Test	Reference value	Day 1	Day 11
Thyroid-stimulating hormone (mIU/L)	0.27–4.20	15.0↑	4.4
Thyroglobulin (ng/mL)	1.60–59.90	82.0↑	42.4
Free T3 (pg/mL)	2.30–4.20	1.67↓	3.3
Free T4 (ng/dL)	0.80–1.80	0.62↓	2.09

Lab results were consistent with hypothyroidism with elevated TSH levels of 15 mIU/L (Table 2). Her initial free T3/T4 levels were measured at a suppressed ratio of 1.67/0.62 (Table 2). Levothyroxine was initiated at 50 μg daily to improve thyroid hormone levels.

The patient was admitted with IV ondansetron, acetaminophen, and potassium. An IV dosage of ceftriaxone was administered for the patient’s pyelonephritis. A red blood cell transfusion was initiated for the patient’s anemia following her low red blood cell count, hemoglobin, and iron levels (Table 1). A urine culture was ordered.

On the second day of admission, the patient's fever persisted at 101.7 °F. Her hematuria and proteinuria persisted. A cystoscopy with the additional use of ureteral stents was performed for the patient's apparent hydronephrosis to reduce urine concentration within her kidneys. A cystoscope was inserted through her urethra into the bladder to find the opening between the ureter and bladder as a ureteral stent was threaded through the cystoscope into the ureter to remove the calcified ureteral stone. This procedure reduces the inflammation of the kidneys by allowing the urine to flow out steadily. Adequate analgesia and intravenous fluids were provided for pain reduction. A combination of remdesivir and azithromycin was initiated after the patient's COVID-19 antigen test returned positive.

On day 3 of admission, the patient's renal lab tests showed gradual improvement, with an assessment to continue the current treatment. Her temperature max (*T*_max_) showed improvement with a reading of 99.8 °F. Ceftriaxone treatment was continued for the patient's underlying acute pyelonephritis and UTI. Chest X-rays demonstrated signs of pneumonia with atelectasis detected at the basal area.

On day 6 of admission, the patient appeared afebrile with a temperature max (*T*_max_) of 98.9 °F. Her hemoglobin levels remained stable, with a minimum hemoglobin level of 12.1 g/dL. Normal potassium levels of 4.2 mEq/L and sodium of 137 mEq/L were replenished as her thrombocytopenia stabilized with a platelet count of 200,000 per mm³. Real-time multiplex PCR tests were performed for *Yersinia enterocolitica*, *Campylobacter jejuni*, Shigella, and Salmonella, all with negative results. The patient’s diarrhea ceased within six days.

The patient's flank pain improved on day 9 of admission. A reverse transcription PCR for SARS-CoV-2 from a throat sample was performed and returned negative. Her thyroid hormone levels returned to appropriate levels (Table 2) as she recuperated well and was classified to be in reasonable health for discharge on day 11. The patient was instructed to continue a ten-day course of treatment of cefuroxime 500 mg twice daily and azithromycin 500 mg once daily for one week. The need to return to the emergency room was emphasized to the patient if she were to experience any concerning symptoms such as a fever, nausea, vomiting, chest pain, shortness of breath, or any other concerning symptoms. Her attending physician informed her of the need for a close follow-up with her primary care physician in two days. She appeared otherwise optimized for discharge with appropriate outpatient follow-up care.

## Discussion

This case presented a unique combination of bacterial hemorrhagic pyelonephritis and ureteral obstruction due to a ureteral calculus in a patient with a history of hypothyroidism diagnosed with SARS-CoV-2 infection. As evidence of the bacteremia and ureteral stone were the direct causes of the patient’s subsequence pyelonephritis and hydronephrosis, this unique presentation focuses on the understudied correlation between COVID-19 and renal injury. This paper is among the first to provide insight into a possible association between endocrinological disorder and COVID-19 regarding the decrease of thyroid hormone levels following exposure to SARS-CoV-2. This occurrence warrants a further review of the understudied relationship between SARS-CoV-2 infection and thyroid hormone alterations that can pose as a risk factor for the pathogenesis of renal injury. There is a need to look further into this relationship and how patients with preexisting thyroid diseases may become a risk factor.

With subacute thyroiditis being a well-documented sequela of COVID-19 infection, most cases have described COVID-19 infection causing symptoms of hyperthyroidism, contrary to this patient’s presentation and others with hypothyroidism [8]. The atypical observation of no tenderness to palpation of the thyroid gland is noteworthy. Recent reports and editorials have suggested a possible connection between deregulated thyroid disorders and SARS-CoV-2 [9]. To avoid misdiagnosis, physicians must examine patients with atypical COVID-19 presentations who potentially display altered levels of thyroid hormones. In addition, to be familiarized with known associated comorbidities and risk factors. A study found that 5.4% of COVID-19 patients were hypothyroid, 90% were 50 years of age or older, with the majority of the patients were female [10]. The destruction of the follicular cells by cytokine storm followed by the thyroid’s innate ability to produce T3/T4 subsequently leads to hypothyroidism. Classic findings consistent with hypothyroidism include elevated levels of TSH with low T3/T4; similar results were observed in this patient (Table 2) [8]. When assessing a patient with hypothyroidism, a thorough history is critical towards establishing it as a potential sequela to COVID-19 infection. This patient denied any history of radiation exposure or prior thyroid surgeries. The diagnosis of both COVID-19 and pyelonephritis required imaging amid the atypical presentations and delayed response to treatment. Chest X-rays that displayed patchy high-density opacities in the patient’s right inftrahilar region (Figure 3) were consistent with abnormal CT findings found in the literature that had a >90% sensitivity for COVID-19 [11].

The dysregulation of the immune system that is frequently seen in patients with hypothyroidism increases the risk for infection, particularly the adverse outcomes of SARS-CoV-2 [10]. The secretion of T3 and T4 significantly impacts the development of kidney diseases by affecting the hypothalamus-pituitary-thyroid axis. This development risks the decrease in T3 levels, the most common laboratory finding of subclinical hypothyroidism in CKD patients [12]. The known association of hypothyroidism with reduced renal plasma flow (RPF) and GFR puts this patient at risk for decreased renal sodium reabsorption and renal ability to dilute urine. The decline in GFR increases the prevalence of subclinical hypothyroidism from 7% to 17.9%, as demonstrated by a study in individuals whose GFR had decreased from ≥90 mL/min to 60 mL/min [12]. A similar finding was observed in this patient with reduced T3 and GFR (Table 2). Hyponatremia was seen in this patient (Table 2). An increase in serum creatinine concentration and hyponatremia are frequently observed in patients with primary hypothyroidism [13].

The timeline between the patient's COVID-19 symptoms and unique hemorrhagic pyelonephritis would establish the SARS-CoV-2 infection of the kidney and urothelium responsible for the bleeding in the patient's urinary tract to the presence of E. coli bacteremia and a ureteral stone to cause obstruction. This association would be adequately established by various methods applied for viral detection in the kidneys, significantly demonstrating the presence of SARS-CoV-2 in patients with COVID-19. The claim of whether this patient’s COVID-19 could have been an underlying cause of the severity of her pyelonephritis could not be established appropriately due to the lack of use of any viral detection methods or renal biopsies to support the association. Early post-mortem kidney biopsies and urine assays using ELISA transcriptional analysis would improve monitoring of SARS-CoV-2 infectivity in the kidneys. Currently, the presence of SARS-CoV-2 in the kidney has been mainly post-mortem, which makes it challenging to detect viral presence as it may be too late after death [6]. Therefore, autopsy studies of patients who died from severe COVID-19 infection are far from ideal, yet represent the majority of reported patients. There are limited studies found where kidney biopsies were completed in the early course of COVID-19-associated kidney disease.

Despite recent reports of renal injury through sepsis or vascular occlusion, there are limited reported relationships between pyelonephritis and ureteral complications caused by SARS-CoV-2 infection [14]. Analysis of previous COVID-19 cases has provided several explanations regarding the development of the disease. Tissue damage to the patient's thyroid gland and its follicular architecture would increase susceptibility to COVID-19 infection with evidence of endocrinological disruption. Interestingly, the receptor for angiotensin-converting enzyme II (ACE2) is expressed on the thyroid parenchyma's follicular cells, the kidney's tubular cells, and the urothelial cells of the bladder, the unique receptor utilized by the SARS-CoV-2 virus to enter human cells. Host cell entry of the virus through the facilitation of the spike (S) protein and transmembrane serine-protease-2 (TMPRSS2) would place the thyroid gland, kidney, and urinary system as high potential targets of direct viral injury. Similar to our patient with a history of hypothyroidism, abnormalities in a patient's thyroid status may impact multiple organ systems, with disorders such as kidney dysfunction. Patients with these conditions and poorly controlled thyroid disorders are at an increased risk of COVID-19 infection [15]. A case series conducted by Cheng et al. showed that 15.5% of patients show evidence of kidney injury on presentation, with 3.2% developing acute kidney injury during hospitalization [16]. Direct and indirect renal injury associated with SARS-CoV-2 via the ACE2 receptor predisposes COVID-19 patients to decreased GFR and renal hypoperfusion. Such an inflammation would predispose a COVID-19 patient to a state of relative immunosuppression and be more prone to severe bacterial pyelonephritis [14].

The finding of a urinary tract infection had caused the patient’s episode of hematuria and the progressive formation of the ureteral stone at the ureterovesical junction. A meta-analysis by Zhang et al. found that 2.4% of every spontaneous renal hemorrhage case was due to infection [17]. As a result, it was stated that this patient had experienced a hemorrhagic sequence and bacterial infection of the urothelium to cause the growth of ureteral calculus and subsequent infiltration of the renal parenchyma. The patient’s urinary culture was positive for E. coli which would support this occurrence with the decline of GFR and subsequent fever. 

Analysis of viruria detected by the use of sensitive methods would provide quintessential information by assessing early-stage SARS-CoV-2 invasion at the site of detection of the kidneys for patients with COVID-19. Studies have shown successful detection of SARS-CoV-2 protein by immunofluorescence (IF), protein mass spectrometry (MS), immunohistochemistry (IHC), and real-time PCR (RT-PCR) [6]. However, technical aspects would need to be considered as these methods require a certain degree of preserved tissue to detect the virus. This requirement is limited due to autolysis in post-mortem samples, resulting in the rise of false-negative results [6]. The use of plaque assay with biopsy material would provide sufficient evidence of kidney invasion by SARS-CoV-2, as demonstrated by the study conducted by Braun et al. to successfully isolate the virus by plaque assay from an autopsied kidney tissue sample [18].

Endocrinological thyroid disorders' impact on COVID-19 patients is currently understudied as epidemiological evidence of comorbidities associated with worsened outcomes is well-established. Published studies have stated that susceptibility to infection is decreased in patients with well-managed hypothyroidism [19]. Therefore, patients with known thyroid diseases are encouraged to maintain their use of thyroid medications to reduce their risk for thyroid dysregulation, which may worsen their outlook for COVID-19 [19].

## Conclusions

We present the case of a 62-year-old female with a history of hypothyroidism who presented with a unique manifestation of acute bacterial hemorrhagic pyelonephritis followed by hematuria after testing positive for COVID-19. The atypical presentations of both COVID-19 infection and hemorrhagic pyelonephritis seen in this case emphasize the importance of conducting a thorough history-taking of any pre-existing thyroid or endocrinological disorders as risk factors for COVID-19, as well as the utilization of imaging to aid in formulating differential diagnoses for any patient displaying atypical presentations. The novel association of hemorrhagic pyelonephritis and COVID-19 is presently understudied in the setting of comorbid hypothyroidism; thus, it could not be established in this study due to the lack of a renal biopsy conducted. We suggest the use of early post-mortem kidney biopsies and urine assays using ELISA transcriptional analysis to help improve the monitoring and understanding of SARS-CoV-2 infectivity towards the kidney. The additional monitoring of thyroid function tests during the acute and convalescent stages of COVID-19 infection until normal thyroid function is restored is recommended.
